# Awareness of Pregnancy Screening for Group B Streptococcus Infection Among Women of Reproductive Age and Physicians in Jeddah, Saudi Arabia

**DOI:** 10.7759/cureus.18765

**Published:** 2021-10-14

**Authors:** Yara Alamri, Samera Albasri, Ghalia H Abduljabbar, Hajar Alghamdi, Albatool M Balkhair, Rawan AlAam

**Affiliations:** 1 Obstetrics and Gynecology, King Abdulaziz University Hospital, Jeddah, SAU

**Keywords:** women, physicians, knowledge, awareness, screening, pregnancy, infection, group b streptococcus

## Abstract

Background

Group B Streptococcus is part of the normal flora of the female urogenital tract and rectum. Vaginal colonization and transmission of this bacteria during delivery can lead to neonatal life-threatening complications, which can be prevented by screening and the administration of intrapartum antibiotics. This study’s aim was to assess the level of awareness of antenatal screening of Group B Streptococcus among women and physicians in Jeddah, Saudi Arabia.

Methods

A cross-sectional study using an online survey from a previously published study was distributed among 767 participants in Jeddah from June to August 2020. The participants were family medicine or obstetrics and gynecology physicians and women of reproductive age.

Results

Our results revealed a good level of knowledge of the physicians, although almost half of them reported the need for training to correctly perform screenings. The level of the women’s knowledge was relatively poor, their mean knowledge was 50.7%, and the majority were unaware of this infection (85.3%).

Conclusions

This study found a low level of knowledge of Group B Streptococcus among women of reproductive age and physicians in obstetrics and gynecology and family medicine. These findings confirm the importance of increasing the awareness of Group B Streptococcus among these populations to avoid complications associated with this infection.

## Introduction

Group B Streptococcus (GBS), also known as Streptococcus agalactiae, is an encapsulated gram-positive diplococcus, normally found in the female urogenital tract and rectum. Vaginal colonization of GBS can lead to maternal and neonatal complications, even when it is asymptomatic [1]. Vertical transmission of GBS before or during delivery is a major cause of premature rupture of the membranes, pre-term delivery, and low birth weight. Furthermore, GBS is an important cause of neonatal sepsis and meningitis, which can increase the rates of neonatal mortality and morbidity [2].

Early and late onsets are two different forms of the disease. The most common cause of neonatal GBS disease is an early-onset infection, which usually occurs within the first week of life, whereas late-onset infection occurs anytime up to three months postpartum [1]. The prevalence of maternal GBS colonization varies from 15% to 30% worldwide [3]; however, in Saudi Arabia, different regions have reported a range of 9.2%-31.6% [2].

A universal culture-based screening program and early administration of intrapartum antibiotics were associated with a decrease in the incidence of early-onset GBS neonatal sepsis in the UK [3]. Moreover, early screening for GBS is effective for identifying and treating the infection, thereby preventing mortality and minimizing its rate [4]. Attention to medical, financial, and educational needs are important for increasing the awareness of GBS screening among patients and physicians aiming to prevent postpartum complications from GBS infection [4]. 

An observational study conducted in a university hospital in Istanbul in 2018, on pregnant women’s awareness of routine screenings and treatments for GBS, found their awareness level rose with increasing maternal age [5]. A survey conducted in Ireland in 2019 measuring the awareness of GBS infection among women evaluated their acceptance of prophylactic vaccination. Of those who responded, 35% had no knowledge of GBS, 29% had little information about GBS, 29% knew about its importance in newborns, and 7% had personal experience with GBS [6].

A cross-sectional study conducted at Prince Sultan Military Medical City in Riyadh, Saudi Arabia, measured the awareness level of primary care physicians regarding GBS pregnancy screening. The mean score for the physicians’ knowledge of GBS screening was 74%; 11.2% of their scores indicated poor knowledge, 31.5% indicated a good level of knowledge, and 57.3% of the scores indicated excellent knowledge. The most frequently reported barriers to GBS screening were systems and protocols by 52.8% of physicians, lack of training by 46.1%, and lack of tools by 24.7%. Furthermore, 14% of physicians cited fear of consequences as a barrier, and 16.9% reported other barriers [4]. A study conducted in Al-Madinah in 2018 that assessed pregnant women's knowledge, attitudes, and current practices related to antenatal GBS screening showed low levels of awareness and knowledge about GBS among them [7].

Although GBS awareness is an important topic, few studies have been conducted in Saudi Arabia on it, and none was carried out in Jeddah. Our research goal was to measure the awareness of GBS screening among women of reproductive age and physicians in Jeddah, Saudi Arabia.

## Materials and methods

Study design and setting

This cross-sectional study was conducted at King Abdulaziz University Hospital in Jeddah, Saudi Arabia, from June to August 2020. The hospital’s institutional review board approved the study (Reference No. 429-20).

Participants

Among the 767 individuals recruited for the study, 745 were included; 25 of the 745 participants were family-medicine or obstetrics and gynecology (OB/GYN) physicians, and the other 720 participants were women of reproductive age (20-50 years old). Unmarried women were excluded from the study. Participation in the study was voluntary, and participants’ identities remained unknown. The questionnaire did not require the disclosure of any personal information. After the individual completed the questionnaire, their responses were considered as consent to participate.

Study procedures and questionnaire

An online self-administered survey was used to obtain data from the participants, and the data were sorted using Google Forms. The physicians’ section enquired about their knowledge, attitudes, and practices related to GBS screening tests. The last part assessed GBS screening difficulties from a physician's perspective. This section was validated in a previous study [4]. The women’s section assessed their obstetric history, knowledge, and awareness of GBS screening, previous exposure to GBS screening, and attitudes toward different GBS screening methods. This section was validated in a different study [7]. Both study groups were asked about their demographics at the beginning of the survey.

Scoring methods

Nine items were used to test physicians' knowledge. Items were scored as either correct (with a value of one) or incorrect (with a value of zero). A total score on the knowledge scale < 50% was considered poor, 50% to < 70%=good, and > 70%=excellent. Six true/false and two multiple-choice questions were used to measure women's knowledge regarding GBS infection, mode of transmission, consequences, and management. Three points were awarded for each correct answer and zero was awarded for incorrect answers. The passing score was 75%. One (Yes/No) question was used to evaluate the awareness levels of the participants, and six Yes/No questions were used to assess participants' experiences.

Data analysis

The Microsoft Excel program version 16.15 (Microsoft Corporation, Redmond, WA) was used to collect and organize the data, and the data were analyzed using SPSS version 21 (IBM Corp, Armonk, NY). Frequencies and percentages were used to describe categorical variables while means and standard deviations were used to describe continuous variables. One-way analysis of variance (ANOVA) and logistic regression were used to analyze other variables. P<0.05 was considered statistically significant.

## Results

A total of 745 participants completed the survey, which assessed the level of awareness of GBS infection among women and physicians in Jeddah. Twenty-five of the 745 participants were family medicine or OB/GYN physicians; their ages ranged from 20 to 50 years old, but they were mainly 41 to 50 years, which accounted for 44% of all participants. Saudi physicians completed the survey more often than did non-Saudi physicians, as the percentage of Saudi physicians enrolled in the study was significantly higher (84%). Most of the participants had a bachelor's degree (72%), followed by a master's degree (20%), and a Ph.D. (8%). The majority of physicians were employed; only 16% were unemployed. Their professional titles included staff physician (16.7%), resident (25%), and registrar or senior registrar (8.3%). Table 1 provides an overview of the contributor’s demographic data.

**Table 1:** Participants’ demographic characteristics * GBS – Group B Streptococcus

Characteristic	Measure	Frequency	Percentage (%)	
Physicians’ characteristics				
Age (years)	20-30	7	28	
	31-40	7	28	
	41-50	11	44	
Nationality	Saudi	21	84	
	Non-Saudi	4	16	
Educational level	Bachelor	18	72	
	PhD	2	8	
	Master	5	20	
Employment	Yes	21	84	
	No	4	16	
Professional title	Staff physician	6		25
	Resident	6		25
	Registrar/senior registrar	2		8.3
	Consultant	10		41.7
Women’s characteristics				
Age (years)	20-30	173	24	
	31-40	265	36.8	
	41-50	282	39.2	
Nationality	Saudi	651	90.4	
	Non-Saudi	69	9.6	
Educational level	Elementary	3	0.4	
	Middle school	8	1.1	
	High school	106	14.7	
	Bachelor	525	73	
	Diploma	37	5.1	
	PhD	13	1.8	
	Master	26	3.6	
	Professor	1	0.1	
Employment	Yes	362	50.3	
	No	358	49.7	
Marriage	Yes	670	94	
	No	43	6	
Pregnancy status	Yes	67	9.3	
	No	653	90.7	
Gestational age	Before 30 weeks	33	49.3	
	After 30 weeks	33	49.3	
Delivery	Vaginal	50	74.6	
	Cesarean section	16	23.9	
Heard about GBS	Yes	170	23.6	
	No	550	76.4	

**Figure 1:**
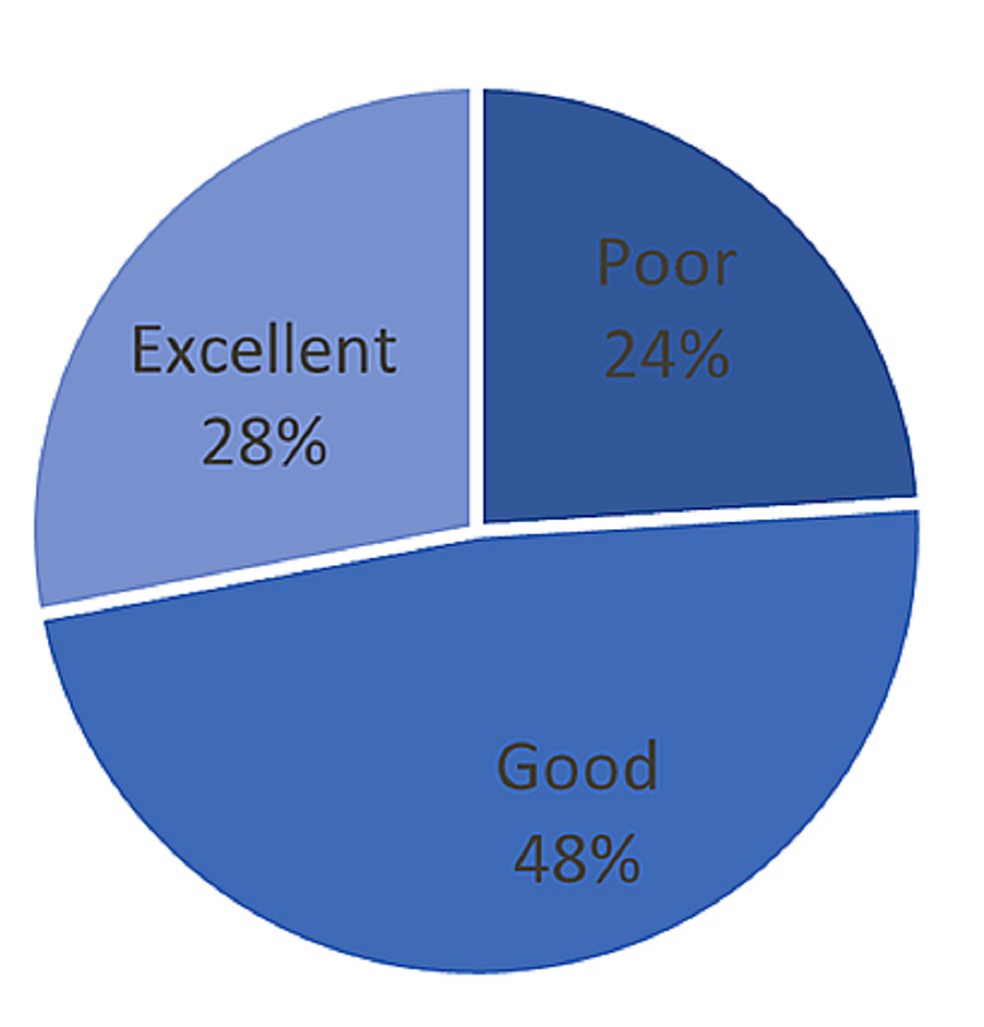
Physicians' knowledge level

Physicians’ knowledge about GBS screening was tested using nine items in the questionnaire. The percentage of correct responses to the items is presented in Table 2. Six of the 25 physicians had poor knowledge, 12 had good knowledge, and seven had excellent knowledge of GBS screening. Figure 1 shows the physician’s scores regarding GBS knowledge statements. Approximately 72% of the physicians responded correctly to the following two statements: “GBS is the most common infection associated with complications in the fetus” and “GBS can be transmitted by vaginal delivery.” Almost all of the physicians (96%) were aware of GBS screening during the prenatal period, which may have facilitated their initiation of antibiotics during labor.

**Table 2:** Frequencies and percentages of physicians’ correct responses to knowledge-related statements * GBS - Group B Streptococcus

Statement	Correct N (%)	False N (%)
GBS is the most common infection associated with complications in the fetus.	18 (72)	7 (28)
GBS can be transmitted to newborns by vaginal delivery.	18 (72)	7 (28)
GBS can cause serious complications to the newborn.	19 (76)	6 (24)
GBS can be asymptomatic during pregnancy.	24 (96)	1 (4)
GBS carrier status is prevalent among pregnant women.	20 (80)	5 (20)
GBS screening can decrease the prevalence of neonatal complications.	19 (76)	6 (24)
GBS screening during the prenatal period can facilitate the initiation of antibiotics during labor.	24 (96)	1 (4)
Asymptomatic pregnant women need to be screened for GBD	14 (56)	11 (44)
Only patients with a history of GBS infection should be screened.	6 (24)	19 (76)

Five statements were used to assess physicians’ attitudes toward GBS screening (Table 3). A large number of physicians agreed with most of the statements, but three of the 25 physicians disagreed with the statement about advising junior staff to perform GBS screenings. Only 12% of them strongly disagreed with recommending GBS screenings in primary healthcare settings.

**Table 3:** Attitudes of family medicine and obstetrics and gynecology physicians toward Group B streptococcus screening statement * GBS - Group B Streptococcus, OB/GYN – Obstetrics and Gynecology

Statement	Strongly Disagree	Disagree	I don’t know	Agree	Strongly Agree
It is important to screen for GBS infection during pregnancy.	2 (8%)	5 (20%)	1 (4%)	4 (16%)	13 (52%)
You recommend screening for GBS infection during pregnancy.	2 (8%)	5 (20%)	0 (0%)	6 (24%)	12 (48%)
You advise junior staff to screen for GBS infection.	5 (20%)	3 (12%)	2 (8%)	7 (28%)	8 (32%)
You think pregnant women will benefit from GBS infection screening.	3 (12%)	4 (16%)	0 (0%)	6 (24%)	12 (48%)
You recommend screening for GBS in primary healthcare settings.	3 (12%)	4 (16%)	4 (16%)	7(28%)	7(28%)

Table 4 presents the GBS screening practices of the physicians. Approximately 40% of them were never trained to screen for GBS. Almost half of them believed it is important to be trained to screen for GBS infection. Approximately 72% screened for GBS in their clinics, and just 12% reported a fear of complications caused by screening for GBS. Only 44% of the physicians trained their junior staff to perform GBS screenings, and 52% initiated treatment with antibiotics for women with GBS during pregnancy.

**Table 4:** Group B streptococcus screening practices of family medicine and obstetrics and gynecology physicians * GBS – Group B Streptococcus

Question	Yes (%)	No (%)	I don’t know (%)
Have you been trained to screen for GBS infection?	14 (56%)	10 (40%)	1 (4%)
Do you need training to perform GBS infection screening?	12 (48%)	10 (40%)	3 (12%)
Do you screen for GBS infection in your clinic?	18 (72%)	7 (28%)	0 (0%)
Do you fear complications from GBS infection screening?	3 (12%)	20 (80%)	2 (8%)
Do you train junior staff to screen for GBS infection?	11 (44%)	10 (40%)	4 (16%)
Do you give antibiotics for GBS infection during pregnancy?	13 (52%)	7 (28%)	5 (20%)

**Figure 2:**
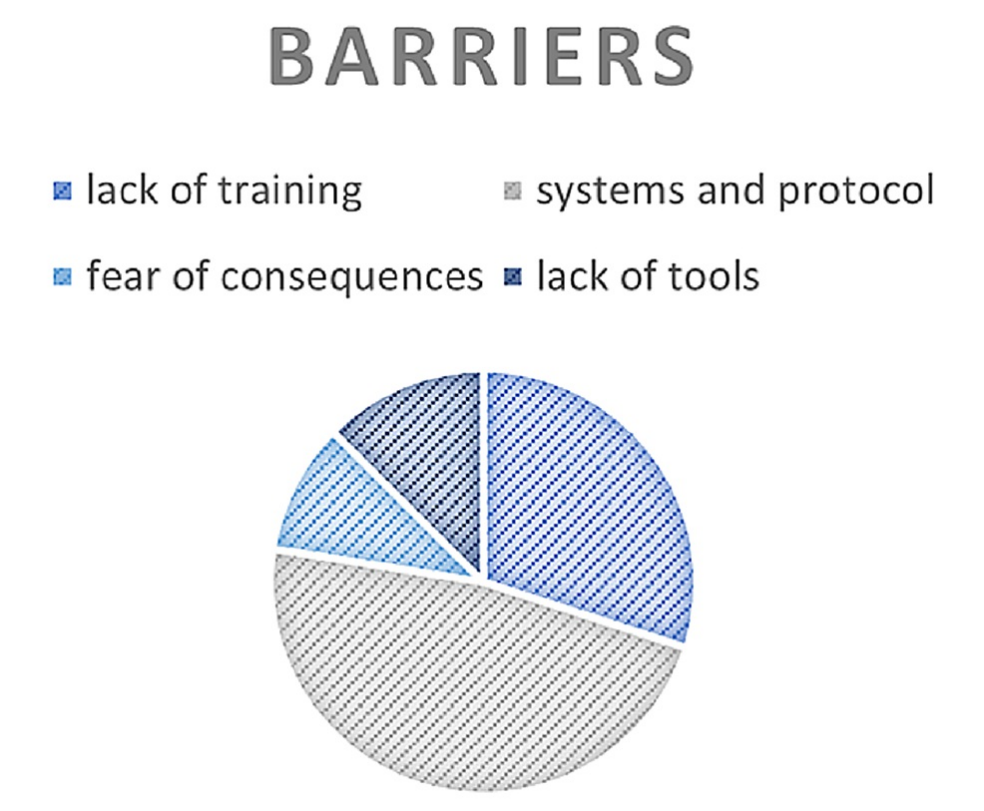
Barriers to Group B Streptococcus screening stated by physicians

Figure 2 reveals the barriers to Group B Streptococcus screening. Physicians stated that systems and protocols were the greatest barriers to GBS screening, and the second barrier was lack of training. Fear of consequences and lack of tools were rarely barriers for them.

One-way analysis of variance was used to compare the means between age groups and the total knowledge scores of the physicians. Differences between all the groups were statistically significant, except for the first and second groups, with p=0.811.

**Figure 3:**
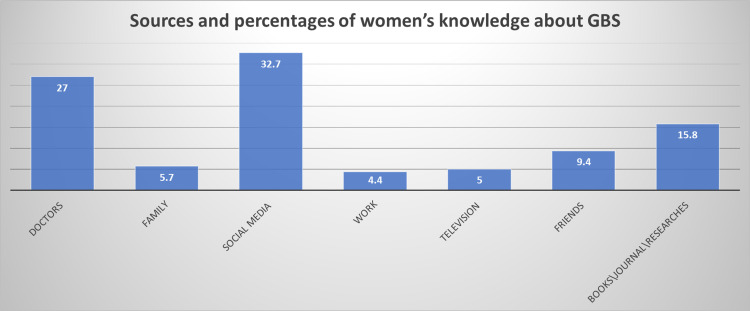
Sources and percentages of women’s knowledge about Group B Streptococcus

The remainder of those who completed the survey were women (720); 39.2% comprised the maximum age group, and 9.6% comprised the minority of non-Saudi participants. All but 11 participants were highly educated. Their socio-demographic data and obstetrics history are presented in Table 1. Nearly all the women were married, and only 9.3% of them were pregnant. The reports of their gestational age (GA) were distributed equally before and after 30 weeks. The largest percentage of women planned to deliver vaginally (74.6%). Most of the women who completed the survey had never heard of GBS during their lifetime (76.4%), and those who had heard of GBS acquired their knowledge from social media. Figure 3 shows other sources of information and their percentages.

Table 5 illustrates the levels of knowledge of GBS among the women in the study. Their mean knowledge score was 50.7% (SD=29.5) and mostly ranged from 50% to 75%. Approximately 59.2% of the women incorrectly agreed that meningitis is a GBS complication, and half of them believed that GBS is a sexually transmitted disease (50.8%). Unfortunately, 45.3% were aware that a mother who is a GBS carrier could not give birth through a normal vaginal delivery, 28.3% stated that carrier mothers cannot breastfeed, and 72.5 % knew that a GBS carrier should take antibiotics during pregnancy.

**Table 5:** Women’s responses to Group B streptococcus knowledge statements and questions * GBS - Group B Streptococcus

Question	Yes N (%)	No N (%)
Is GBS a leading cause of serious blood infections?	366 (50.8)	354 (49.2)
Is GBS a leading cause of meningitis?	294 (40.8)	426 (59.2)
Is GBS a sexually transmitted infection?	366 (50.8)	354 (49.2)
Can GBS be transmitted to a newborn during delivery?	430 (59.7)	290 (40.3)
Is it true that a mother who is a GBS carrier cannot give birth through a normal vaginal delivery?	326 (45.3)	394 (54.7)
Is it true that a mother who is a GBS carrier cannot breastfeed?	204 (28.3)	516 (71.7)
Is the risk of GBS being passed from mother to baby highest (during, before, or after) delivery?	413 (57.4)	307 (42.6)
If a woman carries GBS during pregnancy, when should she receive antibiotics?	522 (72.5)	198 (27.5)

Furthermore, we evaluated the awareness of and past exposure to GBS screening, as shown in Table 6, using frequencies and percentages. Approximately 85.3% of the women in our study were not aware of GBS and 97.5% of them had never had GBS. During their current pregnancies, 86.6% were not informed of the GBS risk assessment. Among the physicians in our study, gynecologists scored the highest (11.9 %) on the item related to informing patients of the risk for GBS during pregnancy while the scores of nurses and other sources of information ranked second (1.5%). Unfortunately, 79.1% of the pregnant women had never been informed of GBS screening assessments.

**Table 6:** Women’s awareness of and past exposure to Group B streptococcus screening * GBS - Group B Streptococcus

Item	Measure	N (%)
Are you aware of GBS? (N=720)	Yes	106(14.7)
	No	614(85.3)
Have you ever had GBS?	Yes	18(2.5)
	No	702(97.5)
During current pregnancy, were you informed of GBS risk assessment?	Yes	6(9)
	No	58(86.6)
Who informed you of GBS risk for pregnant women?	Gynecologist	8(11.9)
	General practitioner/family medicine physician	0(0)
	Nurses/other sources	1(1.5)
	Never been informed	53(79.1)
Are you aware of the available risk screening for GBS during labor?	Yes	6(9)
	No	61(91)
Have you ever been requested to undergo GBS testing?	Yes	52(7)
	No	601(92)
Who requested you to undergo GBS testing?	Gynecologist	49(94.2)
	General practitioner/family medicine physician	1(1.9)
Did you undergo the requested GBS testing?	Yes	46(88.5)
	No	5(9.6)
When were you tested/requested to undergo GBS testing during your pregnancy?	Before or through 30 weeks	14(26.9)
	After 30 weeks	20(38.5)
	I don’t remember	18(34.6)
Were you informed of the GBS testing result?	Yes	44 (84.6)
	No	8(15.4)

The results of the multivariate logistic regression in Table 7show that employment and sources of information were negatively associated with women’s GBS knowledge (“Is Streptococcus B bacteria the main cause of meningitis?”).

**Table 7:** Results of the logistic regression of women’s demographic and clinical characteristics with Group B Streptococcus knowledge * GBS - Group B Streptococcus, GA - Gestational age, CI - Confidence interval, OR - odds ratio, No - number. † P<0.05 is statistically significant

Demographic and clinical characteristics	B	95% CI for the OR		P-value*
		Lower	Upper	
Constant	4.239			0.013
Age	-0.356	0.490	1.001	0.051
Educational level	0.102	0.865	1.416	0.419
Employment (yes)	-0.747	0.306	0.733	0.001
Are you pregnant?	1.703	1.638	18.396	0.006
If yes, what is the GA?	-0.021	0.390	2.463	0.965
What is the method of delivery?	0.223	0.445	3.508	0.672
No. of pregnancies	0.041	0.833	1.302	0.721
No. of deliveries	0.006	0.781	1.296	0.964
Past exposure to GBS	0.462	0.338	7.459	0.558
GBS-knowledge score	-0.443	0.317	1.302	0.219
Have you heard about GBS?	-0.042	0.326	2.819	0.939
Sources of information	-0.582	0.358	0.874	0.011

## Discussion

Infection of the mother and her fetus with GBS during delivery is serious, causing multiple life-threatening complications. The purpose of this study was to evaluate awareness levels of women and physicians about GBS infection screening. Our results showed that 48% of the physicians had a good level of knowledge in contrast to 28% who had excellent knowledge. These findings could be due to a lack of training in Jeddah, as 40% of our participants reported they had never been trained to screen for GBS. This percentage is far below that found in a study conducted in Riyadh (66.3%) [4]. A possible reason for this difference is the limited number of post-graduate training centers and trainers in Saudi Arabia [8].

Furthermore, age has a strong influence on physicians’ knowledge level, as the growing number of years of experience is likely to be accompanied by an increase in their knowledge base. The results of this study support evidence found in previous investigations [4]. Likewise, another study confirmed an association of age with a good level of knowledge among pregnant women [5].

With respect to the physicians’ attitudes toward GBS screening, a few disagreed with the importance of performing GBS screenings during pregnancy, and some of them stated that pregnant women would not benefit from it. However, these responses are discouraging, as both the Centers for Disease Control and Prevention and the American College of Obstetricians and Gynecologists Committee recommend universal culture-based screening along with intrapartum antibiotics to prevent early-onset GBS disease [9-10].

In reviewing the practices of physicians, a plurality stated a need for training to perform GBS infection screening. The greatest proportion of doctors recommended and performed screenings of their patients in the clinic. What is surprising is that the percentage of screenings in our region (72%) was much higher than that in Riyadh (21.3%) [4], which might be related to the lack of training reported by 66.3% of the physicians.

Although a large number of physicians in our study stated that they were not worried about screening complications, some of them did not provide training for the junior staff to perform screenings, which is consistent with the results reported in the Riyadh study [4]. A possible explanation for this finding is the systems and protocols that the physicians identified as the main barriers. Hence, consistent with the study conducted in Riyadh, we endorse the application of internal guidelines and a universal testing policy in all healthcare settings to eliminate the obstacles that physicians frequently encounter [4].

More than half of the physicians in the present study reported giving antibiotics for GBS infection during pregnancy, which confirms the role of antibiotics. A study in the United States estimated that 200 neonatal deaths and 3900 early-onset GBS infections were prevented in 1998 by using intrapartum antibiotics, which decreased the incidence of invasive GBS disease by 21% [11]. Furthermore, research conducted in Spain in 2019 revealed that one of the antibiotics used effectively eradicated GBS from the intestinal and vaginal tracts of pregnant women [12].

The women’s mean knowledge score was 50.7%, which was lower than that of the study conducted in Al Madinah (58.8%) [7]. The lower score might be because 86.6% of them had never been informed of GBS risks during pregnancy. Moreover, our study found that 23.6% of the women heard about GBS while they were in the UK, and 30% never heard about it [13]. Hence, it could conceivably be hypothesized that the higher knowledge level could be attributable to the existence of national guidelines in the UK [12]. These results reflect those of Chow (2011) who also found that only 36% of the pregnant women in Hong Kong had heard about GBS infection [14]. The results of another investigation supporting the work of studies in this area found that 35% of women in Ireland had never heard about GBS [6]. A smaller percentage (30%) reported a similar lack of awareness in Al Madinah, Saudi Arabia [7].

The results of our study show that women generally are not aware of GBS (85.3%). We believe the reason for this finding is that many physicians do not inform their patients about the GBS risk assessments, which could be explained by their reported need for training to perform GBS screenings. Another possible explanation for this lack of awareness is the large number of participants who had never had this infection (97.3%), which is a large number given the reported rate of GBS colonization (9.2%-27.6%) during the third trimester among pregnant women [15]. A comparison of the findings with those reported in studies conducted by Alshengeti et al. and Youden et al. confirmed a low level of GBS awareness among women [7,16]. In addition, only 7% of women have been requested to undergo GBS testing by their gynecologists, whereas 81.7% of pregnant women in Poland have been tested [17]. These vastly different percentages are attributable to the existing protocol in Poland’s Kielce Hospital, requiring midwives to collect a swab for GBS screening if a pregnant woman has no clear tests [18].

Limitations

This study has some limitations. Firstly, due to the circumstances of the coronavirus disease 2019 (COVID-19) pandemic, we were unable to communicate with patients in person during the data collection period to inform them of whether or not some of the questions were dependent on some of their answers. Therefore, their responses that seemed random or were illogical were excluded later. Secondly, the physicians’ increased workload and hours during the pandemic might be the cause of the study’s small sample size; thus, our research does not represent the general population. Extensive studies should be conducted in Jeddah to ensure a sample representative of the populations analyzed. Additionally, all physicians should be interviewed to gather more information about the small number of GBS screening requests. Thirdly, the study’s cross-sectional design is more susceptible than other designs to recall bias of participants regarding GBS screening.

Recommendations

Firstly, training programs are needed to enhance the GBS screening practices of physicians. Secondly, further research is needed to develop an evidence-based universal guideline for GBS screening to improve the womens' and physicians’ knowledge of screening. Educational programs and campaigns should also be considered to increase the public’s health awareness. Thirdly, we suggest that physicians counsel women during the antenatal period regarding GBS infection. Finally, more healthcare centers and internal policies are needed to improve the training and practices of physicians.

## Conclusions

The majority of the physicians in this study reported that they needed training to perform GBS screening. For this reason, more health care centers and internal policies are needed to improve physicians’ training and practices. However, the women's knowledge and awareness scores ranged from 50% to 75%. Thus, the contribution of this study is to confirm the lack of knowledge and awareness of GBS among women in Jeddah, Saudi Arabia. In summary, increasing the awareness level of GBS among the general population and hospital staff is crucial for the avoidance of complications associated with GBS infection.

## References

[1] Musleh J, Al Qahtani N (2018). Group B Streptococcus colonization among Saudi women during labor. Saudi J Med Med Sci.

[2] Khan MA, Faiz A, Ashshi AM (2015). Maternal colonization of group B streptococcus: prevalence, associated factors and antimicrobial resistance. Ann Saudi Med.

[3] Gopal Rao G, Nartey G, McAree T (2017). Outcome of a screening programme for the prevention of neonatal invasive early-onset group B Streptococcus infection in a UK maternity unit: an observational study. BMJ Open.

[4] Almohaimeed M, Al-Arfaj G, Kofi MA (2019). Awareness of primary care physicians about pregnancy screening of Group B Streptococcus infection at Prince Sultan Military Medical City, Riyadh, Saudi Arabia. Int J Adv Community Med.

[5] Kutlu R, Uzun L, Karaoğlu N, Görkemli H (2020). Awareness of pregnant women about routine applied screening tests and supportive treatments in a university hospital. Istanbul Med J.

[6] McCormack S, Thompson C, Chathasaigh CN (2019). GP254 maternal awareness, acceptability and willingness towards group B streptococcus (GBS) vaccination during pregnancy. Arch Dis Child.

[7] Alshengeti A, Alharbi A, Alraddadi S, Alawfi A, Aljohani B (2020). Knowledge, attitude and current practices of pregnant women towards group B streptococcus screening: cross-sectional study, Al-Madinah, Saudi Arabia. BMJ Open.

[8] Al-Khaldi Al-Khaldi, Al-Meqbel Al-Meqbel, Al-Shmmari Al-Shmmari (2014). Challenges facing postgraduate training in family medicine in Saudi Arabia: patterns and solutions. Journal of Health Specialties.

[9] Verani JR, McGee L, Schrag SJ (2010). Prevention of perinatal group B streptococcal disease: revised guidelines from CDC. https://www.cdc.gov/Mmwr/preview/mmwrhtml/rr5910a1.htm.

[10] (2018). Quality of life and sexual function 2 years after vaginal surgery for prolapse: second correction. Obstet Gynecol.

[11] Schrag SJ, Zywicki S, Farley MM (2000). Group B streptococcal disease in the era of intrapartum antibiotic prophylaxis. N Engl J Med.

[12] Martín V, Cárdenas N, Ocaña S (2019). Rectal and vaginal eradication of Streptococcus agalactiae (GBS) in pregnant women by using Lactobacillus salivarius CECT 9145, a target-specific probiotic strain. Nutrients.

[13] Chow TY, To WW, Chan DL (2013). Knowledge and attitudes of Hong Kong pregnant women on group B Streptococcus screening. Hong Kong J Gynaecol Obstet Midwifery.

[14] McQuaid F, Jones C, Stevens Z (2016). Factors influencing women's attitudes towards antenatal vaccines, group B Streptococcus and clinical trial participation in pregnancy: an online survey. BMJ Open.

[15] S. A. Al-Suleiman, I. Farrag, T.-D. Kingsley, S. A. Uduman & M. I. Al-Mouzan (1991 (2009). Third trimester colonisation and treatment of group B β-haemolytic streptococcus among obstetric patients in the eastern province of Saudi Arabia. J Obstet Gynaecol.

[16] Youden L, Downing M, Halperin B, Scott H, Smith B, Halperin SA (2005). Group B streptococcal testing during pregnancy: survey of postpartum women and audit of current prenatal screening practices. J Obstet Gynaecol Can.

[17] Bąk B, Sikorski M, Woźniak A (2016). Demographic conditioning of awareness of prophylaxis against Streptococcus agalactiae (GBS) infections among parturients. Stud Med.

[18] Sibilska MA, Szymankiewicz MA, Gadzinowski JA, Bręborowicz GH, Szmyt HA (2014). Presence of group B streptococci (GBS) in newborns in the aspect of intrapartum prophylaxis [Article in Polish]. Perinatol Neonatol Ginekol.

